# Immunogenicity of a plasmid DNA vaccine encoding G1 epitope of bovine ephemeral fever virus G glycoprotein in mice

**DOI:** 10.4102/ojvr.v85i1.1617

**Published:** 2018-08-28

**Authors:** Reza Pasandideh, Masoud Reza Seyfi Abad Shapouri, Mohammad Taghi Beigi Nassiri

**Affiliations:** 1 Department of Animal Science, Khuzestan Agricultural Sciences and Natural Resources University, Ahvaz, Islamic Republic of Iran; 2 Department of Pathobiology, Shahid Chamran University of Ahvaz, Islamic Republic of Iran

## Abstract

The aim of this study was to investigate the immunogenicity of a plasmid deoxyribonucleic acid (DNA) vaccine encoding the G1 epitope of bovine ephemeral fever virus (BEFV) G glycoprotein in mice. A plasmid DNA carrying the G1 gene was constructed and designated as pcDNA3.1-G1. The expression of the target gene was confirmed in human embryonic kidney 293 (HEK 293) cells transfected with pcDNA3.1-G1 by indirect immunofluorescent staining. Immunisation experiments were intramuscularly carried out by vaccinating 6-week-old female mice in four groups, including the pcDNA3.1-G1 construct, pcDNA3.1 (+) plasmid alone, BEF-inactivated vaccine and phosphate-buffered saline (PBS) (1X) three times with 2-week intervals. Fourteen days after the last immunisation, the animals were bled and the resulting sera were tested for anti-G1-specific antibodies by immunoblotting analysis, indirect enzyme-linked immunosorbent assay (ELISA) and virus neutralisation (VN) test. Serological assays showed that the pcDNA3.1-G1 construct expressing G1 protein was able to elicit specific antibodies against this antigen. Virus neutralisation test showed that pcDNA3.1-G1 could induce anti-BEFV-neutralising antibodies in mice. Our findings indicated that a new dimension can be added to vaccine studies for bovine ephemeral fever (BEF) using eukaryotic expression plasmids encoding the G1 antigen in the future.

## Introduction

Bovine ephemeral fever (BEF) is a viral disease of cattle and water buffalos seen in Africa, the Middle East, Australia and Asia. Infected cattle can show a wide spectrum of clinical signs, including a sudden onset of fever (41 °C – 42 °C) with loss of appetite, increased breathing and heart rate, stiffness, lameness, cessation of rumination and constipation (Walker 2005; Zheng et al. 2009). Bovine ephemeral fever is an economically important disease, which can be spread rapidly and lead to considerable losses in the cattle industry, through reduced milk production in dairy herds, loss of condition in beef cattle and the immobilisation of draught animals (Aziz-Boaron et al. 2013; Walker 2005). Bovine ephemeral fever is caused by BEF virus (BEFV) and transmitted through mosquitoes or biting midges. Bovine ephemeral fever virus is classified as a member of the genus *Ephemerovirus* in the family Rhabdoviridae. Bovine ephemeral fever virus has a bullet-shaped morphology, contains a 14.9 kb single-stranded, negative-sense ribonucleic acid (RNA) genome, which encodes five structural proteins, including a nucleoprotein (N), a polymerase-associated protein (P), a matrix protein (M), a large RNA-dependent RNA polymerase (L) and a glycoprotein (G) spanning the viral envelope and a non-structural glycoprotein (GNS). G protein is the main protective antigen of the virus and the target of anti-BEFV-neutralising antibodies and harbours five distinct antigenic sites – G1, G2, G3a, G3b and G4 – on its surface (Cybinski et al. 1992; Dhillon et al. 2000; Kongsuwan et al. 1998). Epitope-G1 is a linear site (Y^487^–K^503^) in the C-terminal region of the ectodomain (Trinidad et al. 2014) that only reacts with sera against BEFV, but other antigenic sites have cross-reactions with the sera against the related viruses besides BEFV (Yin & Liu 1997).

The prevention and control of BEF infection can be achieved through vaccination and treatment of affected cattle (Aziz-Boaron et al. 2013; Wallace & Viljoen 2005). Various studies have been conducted to develop an efficient vaccine for BEF, including live attenuated, inactivated, subunit G protein-based and recombinant vaccines (Walker & Klement 2015). An effective vaccination has been obtained using the BEFV G glycoprotein split from a semi-purified virus in cattle (Bai et al. 1993). In addition, the BEFV G glycoprotein delivered in recombinant virus vectors has induced specific neutralising antibodies and cell-mediated immune responses in cattle (Hertig et al. 1996; Wallace & Viljoen 2005). Therefore, it appears that the recombinant expressed BEFV G protein may serve as a useful vaccine antigen (Johal et al. 2008). Deoxyribonucleic acid (DNA) immunisation is a promising approach for vaccination by injection of an isolated eukaryotic expression plasmid encoding the antigen (Watts & Kennedy 1999). DNA vaccination has been used successfully to immunise various animal species against many infectious agents and has several advantages over other vaccination approaches (Corr et al. 1996; Fynan et al. 1993; Robinson, Hunt & Webster 1993; Sakaguchi et al. 1996). However, no effort has been made so far regarding the evaluation of the efficacy of a DNA vaccine based on BEFV G glycoprotein against BEF. Hence, the purpose of this study was to investigate the immunogenicity of a plasmid DNA vaccine encoding the G1 epitope of BEF virus G glycoprotein in mice.

## Materials and methods

### Virus, cell lines, bacterial strain and vector

The strain of BEF virus used in this study was procured from Razi Vaccine and Serum Research Institute (Hesarak, Karaj, Iran). Basic local alignment search tool (BLAST) analysis based on G gene sequence showed that this strain had the highest identity with the YHL strain isolated in Japan’s Yamaguchi prefecture in 1966 (Pasandideh et al. 2016). Hamster lung (HmLu-1) cells were used to propagate the BEFV using Roswell Park Memorial Institute (RPMI) medium (Bio Idea, Iran) supplemented with 5% fetal bovine serum (Gibco, UK). Human embryonic kidney 293 (HEK 293) cells were used for plasmid transfection and expression experiments. HmLu-1 and HEK 293 cell lines were received from the National Cell Bank of Iran (NCBI) affiliated with the Pasteur Institute of Iran. DH5α strain of *Escherichia coli* (*E. coli*) (CinnaGen, Iran) and pcDNA3.1 (+) eukaryotic expression vector (Invitrogen, Carlsbad, CA, United States of America [USA]) were used for the cloning and protein expression experiments.

### Construction and preparation of expression vector

The 420 base pairs (bp) fragment of BEFV G1 gene was previously cloned into the pcDNA3.1 (+) vector under the control of the human cytomegalovirus (CMV) promoter using the G1 specific primers G1-fwd-*Kpn*I: 5’-GTGGGTACCGCCACCATGGTGAGAGCTTGGTGTGAATACA-3’ and G1-rev-*Bam*HI: 5’-CATTGGATCCTCACCAACCTACAACAGCAGATA-3’. Then, the pcDNA3.1-G1 construct containing the 420 bp fragment was transfected into HEK 293 cell line to consider protein expression. Finally, the expression efficiency was verified by indirect immunofluorescent staining (Pasandideh et al. 2018). After verification of protein expression, the pcDNA3.1-G1 construct was amplified in *E. coli* DH5α and purified with the Endofree Plasmid Purification Kit (Qiagen, Germany), according to the manufacturer’s instructions, and then used for immunisation of mice.

### Mice and immunisation

Six-week-old female mice of N-MARI strain were intramuscularly inoculated in four groups of five each. Group A mice were immunised with 100 *µ*g/animal of the endotoxin-free pcDNA3.1-G1 construct in an appropriate rate of phosphate-buffered saline (PBS) (1X) in the anterior quadriceps muscle. Group B mice were vaccinated with 200 *µ*L/animal of BEF-inactivated vaccine (Kyoto Biken Laboratories, Japan). Control groups were only inoculated with 100 *µ*L/animal of PBS (1X) or 100 *µ*g/animal of empty pcDNA3.1 (+) plasmid. Immunisation was repeated two more times with 2-week intervals. Fourteen days after the third immunisation, the animals were bled and the resulting sera were tested for anti-G1-specific antibodies by immunoblotting analysis, indirect enzyme-linked immunosorbent assay (ELISA) and virus neutralisation (VN) test. A purified prokaryotic G1 protein with ~18 kDa molecular weight, produced in our previous study, was used as a coating antigen to develop immunoblotting and indirect ELISA in this study.

### Detection of anti-G1-specific antibodies by immunoblotting

Immunoblotting analysis was used to investigate the immunogenicity of pcDNA3.1-G1 and detect anti-G1-specific antibodies in the serum of mice. For this purpose, the 18-kDa prokaryotic G1 protein was electrophoresed on 15% sodium dodecyl sulphate (SDS)-polyacrylamide gel and then transferred to a nitrocellulose membrane by electroblotting at 60 volt (V) for 3 hours. The blotted membrane was blocked by PBS with 0.05% Tween-20 (PBST) containing 5% skim milk overnight at 4 °C and then washed three times with PBST. The nitrocellulose membrane was cut into strips and incubated with the mouse sera diluted 1:20 in PBST containing 5% skim milk for 2 h at room temperature, individually. After washing with PBST, an anti-mouse IgG (H+L)-HRP (Bio-Rad Laboratories, USA) diluted 1:3000 in PBST containing 5% skim milk was added to the strips for 1 h at room temperature. After extensive washing, the strips were dipped in PBS containing H_2_O_2_, 4-chloro-l-naphthol and methanol to view the result of reaction. The colour developing reaction was stopped with distilled water.

### Evaluation of anti-G1-specific antibody titers by indirect enzyme-linked immunosorbent assay

Anti-G1-specific antibody titers in the serum of each immunised mouse were determined by an indirect ELISA. Ninety-six-well immunoplates were coated with the prokaryotic G1 protein diluted 1:200 in carbonate coating buffer (0.5 M NaHO_3_/Na_2_CO_3_, pH 9.3) for 16 h at 4 °C. The final concentration of coating antigen was 0.25 *µ*g/well by calculation. After washing three times with PBST to remove the unbound antigen, the plates were blocked with 300 *µ*L of PBST containing 5% skim milk for 3 h at 37 °C. The plates were washed again with PBST and then incubated with the mouse sera (50 *µ*L/well) diluted 1:100 in PBST containing 5% skim milk for 45 minutes at room temperature. After washing as previously mentioned, an anti-mouse IgG (H+L)-HRP (Bio-Rad) diluted 1:3000 in PBST containing 5% skim milk was added for 45 min at room temperature. The plates were washed four times with PBST and finally 50 *µ*L of the chromogen substrate (tetramethylbenzidine 1%, 0.1 M sodium acetate [pH 6] and H_2_O_2_ 3%) was added to each well and incubated at room temperature in the dark for 10 min. The reaction was stopped by adding 50 *µ*L of chloridric acid (0.1 M) and the absorbance was read immediately at 450 nanometers (nm) by an ELISA spectrophotometer.

### Detection of anti-bovine ephemeral fever virus neutralising antibodies

Mouse sera were tested for the presence of anti-BEFV-neutralising antibodies by VN assay. Briefly, the sera were heat-inactivated at 56 °C for 30 min, and then 50 *µ*L of two-fold serial dilutions of each serum was mixed with 50 *µ*L of 100 TCID_50_ of BEFV in the 96-well tissue culture plate and then incubated for 1 h at 37 °C in 5% CO_2_. After the incubation period, 50 *µ*L of the Vero cell suspension containing 15000 cells was added to each well and the plate was incubated for 4 days at 37 °C in a humidified incubator with an atmosphere of 5% CO_2_. After the incubation, the cells were examined for BEFV-specific cytopathic effects (CPEs) using an Olympus IX71 inverted optical microscope (Olympus Australia, Mt. Waverley, Australia).

### Statistical analysis

SAS software (version 9.1; SAS Institute) was used for the analysis of ELISA data. Least significant difference (LSD) test was used to compare the mean of each group of mice, and a *p*-value of less than 0.01 was considered statistically significant.

### Ethical consideration

The authors declare that the project underwent ethical review and was given approval by an institutional animal care and use committee or by appropriately qualified scientific and lay colleagues. The care and use of experimental animals complied with local animal welfare laws, guidelines and policies. Animal studies have been approved by the appropriate ethics committee of the Khuzestan Agricultural Sciences and Natural Resources University (1/411/1189).

## Results

### Expression of G1 protein by the pcDNA3.1-G1 construct

**FIGURE 1:**
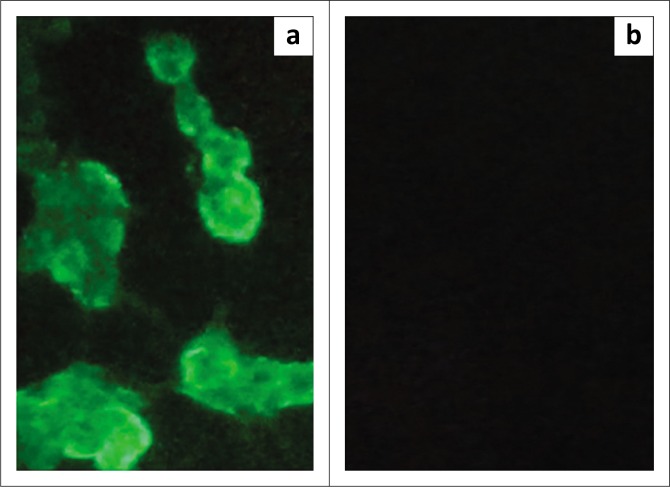
Verification of G1 protein expression in immunofluorescence staining. (a) Image of the transfected human embryonic kidney 293 (HEK 293) cells with the pcDNA3.1-G1 construct obtained by a fluorescence microscope and (b) transfected HEK 293 cells with empty pcDNA3.1 plasmid used as negative control.

The expression of G1 protein by pcDNA3.1-G1 was confirmed using indirect immunofluorescence staining. The observation of intracytoplasmic fluorescence in the transfected cells with pcDNA3.1-G1 in reaction to an anti-G1 monospecific polyclonal antibody, produced in our previous study (Beygi Nassiri, Pasandideh & Seyfi Abad Shapouri 2016), indicated that G1 protein was successfully expressed by pcDNA3.1-G1 in the HEK 293 cells (Figure 1).

### Verification of anti-G1-specific antibodies using immunoblotting

**FIGURE 2:**
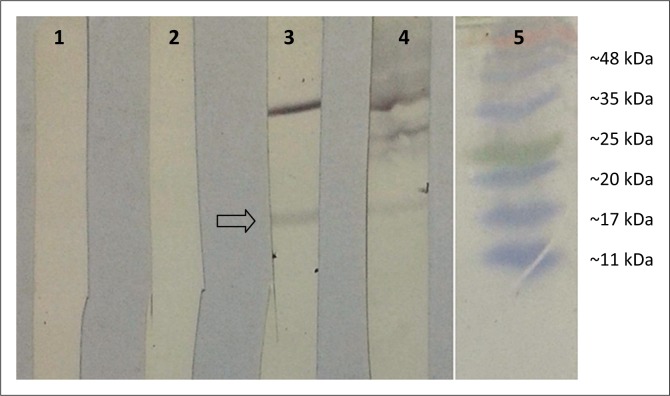
Verification of anti-G1 antibodies against pcDNA3.1-G1 and bovine ephemeral fever (BEF)-inactivated vaccine in reaction to the 18-kDa G1 protein using immunoblotting. Lanes 1–4, respectively, show the membrane strips exposed to serum of inoculated mice with phosphate-buffered saline (1X), pcDNA3.1, pcDNA3.1-G1 and BEF-inactivated vaccine; Lane 5 shows the marker polypeptides.

Immunoblotting analysis was used to evaluate the immunogenicity of the pcDNA3.1-G1 construct in mice. For this purpose, the 18-kDa prokaryotic G1 protein was blotted onto a nitrocellulose membrane and exposed to serum of inoculated mice, separately. As shown in Figure 2, the appearance of a distinct band with an approximate molecular weight of 18 kDa for immunised mice by pcDNA3.1-G1 and BEF-inactivated vaccine indicated that specific antibodies against BEFV G1 protein were induced in these groups.

### Anti-G1-specific antibody titers after DNA vaccination

Two weeks after the last immunisation, anti-G1-specific antibody titers in serum of inoculated mice were assayed using ELISA. It was observed that anti-G1 antibody titers in mice immunised with pcDNA3.1-G1 were significantly higher than those in control groups for plasmid and PBS 1X (*p* < 0.01). The most significant anti-G1-specific antibodies were elicited in mice vaccinated with BEF-inactivated vaccine (*p* < 0.01) (Table 1).

**TABLE 1:** Anti-G1 antibodies produced in mice after intramuscular inoculation of plasmids and bovine ephemeral fever-inactivated vaccine measured by enzyme-linked immunosorbent assay.

Studied group	Mean±SD
PBS (1X)	0.056±0.002*
pcDNA3.1	0.059±0.008*
pcDNA3.1-G1	0.210±0.057**
BEF-inactivated vaccine	0.307±0.073***

Note: The results were expressed as mean ± standard deviation of optical density (450 nm) of each group of five mice.

PBS, phosphate-buffered saline; SD, standard deviation; BEF, bovine ephemeral fever.

* , No significant difference (*p* > 0.05);

**, *** , Significant difference (*p* < 0.01) between **, *** and *.

### Induction of neutralising antibodies against bovine ephemeral fever virus

**FIGURE 3:**
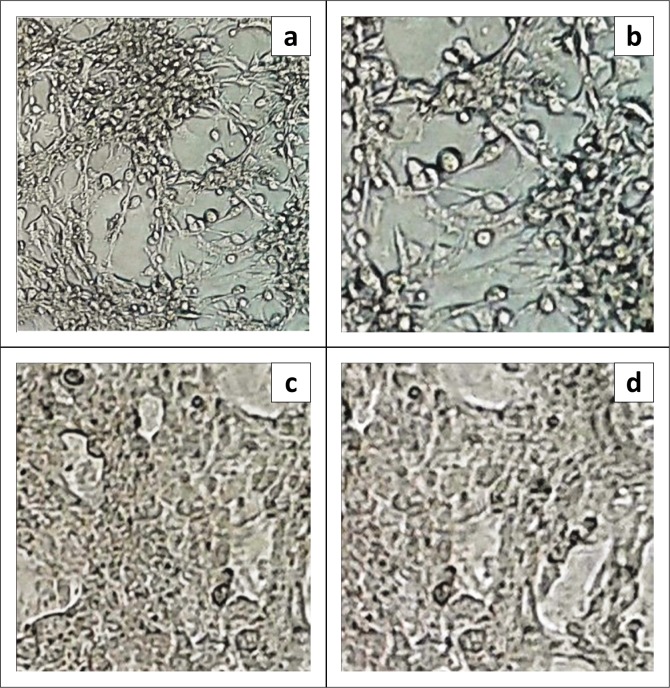
Microscopic examination of the Vero cells for evidence of viral cytopathic effects in virus neutralisation assay. (a and b) The CPEs in the cells treated with the sera collected from control groups for pcDNA3.1 and PBS, respectively; (c and d) the cells treated with the sera collected from the groups immunised with pcDNA3.1-G1 and bovine ephemeral fever (BEF)-inactivated vaccine, respectively.

The presence of anti-BEFV-neutralising antibodies in the sera of immunised mice was investigated by VN assay. The sera collected from mice immunised with pcDNA3.1-G1 and BEF-inactivated vaccine neutralised all the virus activity up to 1:50 dilution and prevented BEFV-specific CPEs from developing in Vero cells (Figure 3). Therefore, our results indicated that immunisation with the pcDNA3.1-G1 construct could elicit neutralising antibody responses against BEF virus. As expected, the CPEs caused by the virus proliferation were observed in the cells treated with the sera collected from the control groups for plasmid backbone and PBS (1X).

## Discussion

Natural BEF infection leads to long-term immunity in affected animals (Mackerras, Mackerras & Burnet 1940). Hence, vaccination can be considered as an effective approach of prevention against the disease. So far, several studies have been developed to produce diverse vaccines for BEF, including live attenuated, inactivated, subunit G protein-based and recombinant vaccines. Live attenuated, inactivated and subunit vaccines are being used in the field (Walker & Klement 2015). However, according to our knowledge, no study has been performed to design a DNA vaccine based on G1 gene for immunisation against BEF and this was the first study in this area. In this study, a eukaryotic expression construct for G1 epitope of BEFV G glycoprotein gene was designed in order to evaluate its immunogenicity and efficacy in mice. Serological assays showed that the pcDNA3.1-G1 construct expressing G1 protein was able to induce specific immunity and produce antibodies against this antigen. However, the anti-G1-specific antibody titers against pcDNA3.1-G1 were significantly lower than those against BEF-inactivated vaccine. It may be for this reason that only an antigenic site of BEFV G glycoprotein gene was used in the pcDNA3.1-G1 construct. Virus neutralisation test showed that pcDNA3.1-G1 could induce anti-BEFV-neutralising antibodies in immunised mice.

In previous studies, it was found that BEFV G protein could induce virus-specific neutralising antibodies and confer passive protection against intracerebral infection of suckling mice (Cybinski et al. 1990) and protect cattle against experimental intravenous BEFV challenge (Hertig et al. 1996; Johal et al. 2008; Uren et al. 1994). The nucleotide and amino acid sequences of the G1 antigenic site of G glycoprotein have been highly conserved among all isolates, except for an amino acid substitution at position 499 for a few strains (Kato et al. 2009; Zheng & Qiu 2012). However, amino acid variations detected in the main neutralisation sites (G1, G2 and G3) of the G protein did not affect the neutralisation properties of these epitopes (Trinidad et al. 2014). High immunogenicity of G glycoprotein and the fact that G1 epitope has been genetically and antigenically conserved among various isolates of BEFV allow the use of G1 as a useful vaccine antigen. Therefore, G1 antigen was chosen for application as a possible DNA vaccine for immunisation of mice in this study. Today, DNA vaccines are widely considered because of many advantages, such as safety, stability, low costs and longer immunogenicity (Porter & Raviprakash 2017).

The induction of anti-BEFV-neutralising antibodies in mice by the pcDNA3.1-G1 construct expressing G1 protein in our research was consistent with the immunogenicity of subunit and recombinant vaccines based on G glycoprotein in previous studies. For example, vaccination using the BEFV G protein split from a semi-purified virus induced a neutralising antibody response and protected 50% of cattle in China (Bai et al. 1993). In Australia, administration of a G protein subunit vaccine with Quil A adjuvant protected 100% of cattle against experimental challenge (Uren et al. 1994). Vaccination with recombinant New York Board of Health (NYBH) strain of vaccinia virus expressing the BEFV G protein could elicit specific neutralising antibodies in cattle, but the protection experiment was inconclusive (Hertig et al. 1996). In the same experiment, four doses of the Neethling strain of lumpy skin disease virus expressing the BEFV G protein induced a specific neutralising antibody and cell-mediated immune responses in cattle but protection failed 10 weeks after the last dose (Wallace & Viljoen 2005). Although various vaccines with different formulations have been developed for BEF, there are few reports about the assessment of the vaccines under conditions in the field and their usage rates are often low. It appears that protective immunity for most of these vaccines continues for a limited period and their efficiency may be insignificant unless additional booster doses are administered at intervals of 6 months to 1 year (Walker & Klement 2015). Therefore, other advanced technologies are needed to decrease the required number of doses and prolong the duration of protection. On the other hand, the cell-mediated responses may also be involved in protection against BEF, especially for the longer-term sequelae that occur in some animals (Della-Porta & Snowdon 1979; Walker & Klement 2015). Regarding the ability of DNA vaccines to induce protective humoral and significant cellular immune responses to the expressed antigens (Khan 2013), it seems DNA vaccination can be an appropriate approach against BEF. However, as found in this study, the eukaryotic expression plasmids encoding the antigens usually induce fewer responses compared to live or inactivated pathogen immunisation (Shah et al. 2011). We suggest the use of genetic adjuvants such as cytokine genes with the G1 antigen to improve the efficacy of the pcDNA3.1-G1 construct in future studies.

## Conclusion

This study demonstrated that the pcDNA3.1-G1 construct could induce immunity and protection against the BEFV in an animal model. Our findings indicated that a new dimension can be added to vaccine studies for BEF using eukaryotic expression plasmids encoding the G1 antigen in the future. Obviously, further studies are needed to improve this type of vaccines and obtain more comprehensive information about their performance in the main hosts.
